# Applying a modified metabarcoding approach for the sequencing of macrofungal specimens from fungarium collections

**DOI:** 10.1002/aps3.11508

**Published:** 2023-02-02

**Authors:** C. Gary Olds, Jessie W. Berta‐Thompson, Justin J. Loucks, Richard A. Levy, Andrew W. Wilson

**Affiliations:** ^1^ The Department of Research and Conservation Denver Botanic Gardens Denver Colorado USA; ^2^ The Department of Integrative Biology University of Colorado Denver Denver Colorado USA

**Keywords:** DNA barcode, fungarium collections, fungi, Illumina sequencing, nrITS2, Sanger sequencing

## Abstract

**Premise:**

Fungaria are an underutilized resource for understanding fungal biodiversity. The effort and cost of producing DNA barcode sequence data for large numbers of fungal specimens can be prohibitive. This study applies a modified metabarcoding approach that provides a labor‐efficient and cost‐effective solution for sequencing the fungal DNA barcodes of hundreds of specimens at once.

**Methods:**

We applied a two‐step PCR approach using nested, barcoded primers to sequence the fungal nrITS2 region of 766 macrofungal specimens using the Illumina platform. The specimens represent a broad taxonomic sampling of the Dikarya. Of these, 382 *Lactarius* specimens were analyzed to identify molecular operational taxonomic units (MOTUs) using a phylogenetic approach. The raw sequences were trimmed, filtered, assessed, and analyzed using the DADA2 amplicon de‐noising toolkit and Biopython. The sequences were compared to the NCBI and UNITE databases and Sanger nrITS sequences from the same specimens.

**Results:**

The taxonomic identities derived from the nrITS2 sequence data were >90% accurate across all specimens sampled. A phylogenetic analysis of the *Lactarius* sequences identified 20 MOTUs.

**Discussion:**

The results demonstrate the capacity of these methods to produce nrITS2 sequences from large numbers of fungarium specimens. This provides an opportunity to more effectively use fungarium collections to advance fungal diversity identification and documentation.

Only 8% of the estimated 2.2 to 3.8 million fungal species worldwide have been documented (Hawksworth and Lücking, 2017). Our ability to address the gap between known and unknown fungal diversity will require new approaches in methodology and technology. Fungaria (herbaria of fungi) are an essential resource that preserve and provide the specimens necessary for investigating and documenting fungal diversity (Andrew et al., 2018). Many fungarium collections consist of macrofungi, which are fungi that produce macroscopic reproductive structures (e.g., mushrooms, puffballs, club fungi, coral fungi, cup fungi, truffles). These fungi represent diverse taxonomic groups predominantly from the subkingdom Dikarya (phyla Ascomycota and Basidiomycota). The specimens from these collections provide opportunities for research into the taxonomy, systematic diversity, evolution, and ecology of fungi in both narrow and broad scopes (Andrew et al., 2018). Despite this, fungaria remain an underutilized resource for exploring questions in fungal biology, in large part because many specimens have incomplete or suspicious identifications. Using DNA sequence data is a logical step toward identifying undescribed fungal specimens; however, there are several challenges associated with generating DNA sequence data from large numbers of specimens, which can be summed up in three parts: scale, sequencing effectiveness, and cost.

The challenge of scale is exemplified by the number of unidentified and misidentified specimens in fungaria. For clarification, we use “unidentified” to indicate “not identified to species” and “misidentified” to mean anything from an incorrect identification to an identification using out‐of‐date taxonomic concepts. The large number of unidentified fungal specimens is a major impediment for examining questions in fungal population genetics, ecology, and distributions. The Mycology Collections Portal (MyCoPortal; https://mycoportal.org/portal/index.php [accessed 25 May 2022]) database of North American fungaria has records for 6,613,990 fungal specimens. Of these, 1,580,404 (24%) remain unidentified. The Global Biodiversity Information Facility (GBIF; https://www.gbif.org) database aggregates records of fungal specimens globally, containing over 7.92 million records (GBIF.org, 2021). Of these, 2.77 million include the specific epithets, suggesting that around 65% of fungal specimens in global collections are unidentified. This problem becomes compounded when we consider the number of misidentified specimens in fungaria. The challenge of scale posed to fungal systematics and taxonomy emphasizes the need to develop tools that aid in the identification of fungarium specimens.

For fungal diversity studies, DNA barcoding has become a necessary first step in species recognition (Lücking et al., 2020). A historical reliance on morphology in fungal systematics has generally produced the underlying species concepts, but molecular analysis provides the finer‐scale resolution necessary for the identification of cryptic species. The DNA barcode for fungi is the nuclear ribosomal internal transcribed spacer (nrITS) region (Schoch et al., 2012), which was selected because of its high PCR amplification success rate coupled with its high interspecific but low intraspecific variation. Sequences of nrITS from many fungal specimens have been shown to be effective at recognizing useful molecular operational taxonomic units (MOTUs) in multiple taxonomic groups (Begerow et al., 2010; Osmundson et al., 2013). These features have helped make nrITS the most common fungal sequence on GenBank (Lücking et al., 2020). Despite this, the vast majority of accepted fungal species do not have a representative nrITS sequence on GenBank (Hawksworth and Lücking, 2017). When this fact is combined with the potentially large number of misidentified specimens in fungaria, the potential for identifying new species from macrofungal specimens becomes too significant to ignore. Fortunately, the challenge facing mycologists who need to efficiently produce DNA barcode data from hundreds to thousands of fungal specimens can be addressed by modern high‐throughput sequencing technologies.

The challenge of sequencing effectiveness touches on how well a technology can produce DNA barcode sequence data of sufficient quality. The Sanger sequencing approach to DNA barcoding fungal specimens has been used for over three decades (White et al., 1990), and many systematic and taxonomic studies currently rely on its effectiveness and practicality (Noffsinger and Cripps, 2021; Vera et al., 2021). Despite Sanger sequencing's long and productive history, there are some significant limitations to its effectiveness. One of these limitations is the inability to sequence degraded DNA obtained from older specimens. Additionally, translating the sequences into usable nrITS data can be complicated by irregular insertions and deletions of nucleotides among ribosomal copies, resulting in frame shifts that are often interpreted as noise in the electropherograms. The issue of scale when producing nrITS data from large numbers of unidentified fungarium specimens quickly runs up against the barrier of cost using the Sanger method; for example, we are charged approximately $4.00 USD per sequence when we outsource Sanger sequencing. Forward and reverse sequence reads are generally produced for redundancy and to span the sequence length from both directions, making the cost of one nrITS contiguous sequence (or contig) per specimen approximately $8.00 USD. Scaling this cost to sequence thousands of unidentified specimens in fungaria quickly makes it prohibitive to perform comprehensive studies in fungal diversity.

High‐throughput sequencing (HTS; e.g., next‐, second‐, and third‐generation sequencing) can address the challenges of scale, effectiveness, and cost when generating DNA barcode sequence data from fungal specimens. The ability to sequence multiple reads from the same sample can be used to validate a sequence and address problems such as amplicon length heterogeneity, which tends to be a challenge with Sanger sequencing. Metabarcoding studies use HTS technology to generate a portion of the fungal DNA barcode to document communities from soil and tissue samples (Nilsson et al., 2019). Given that a single study can produce sequence data representing hundreds to thousands of species, the method has potential for use on numerous fungarium collections. Several versions of HTS produce short reads, and metabarcoding studies that use these versions need to focus on one of two regions in the nrITS: ITS1 or ITS2. The utility of these markers for DNA barcoding can vary among taxa, but the overall differences in phylogenetic signal between the two are considered negligible, and similar results can be obtained from either marker (Bazzicalupo et al., 2013; Blaalid et al., 2013). Of the two, the ITS2 region is slightly more favored for DNA barcoding because of the number of reference sequences available (Nilsson et al., 2008; Lücking et al., 2020) and the complications in targeting ITS1 in some taxa (Lindahl et al., 2013). In terms of cost, approaches such as the Illumina MiSeq Nano (Illumina, San Diego, California, USA) provide 800,000 read pairs for a little over $1000 USD, plus approximately $500 for library quantification, cleaning, and additional PCRs through the sequencing facility (based on costs from the University of Idaho's IIDS Genomics and Bioinformatics Resources Core [GBRC]). As Illumina sequencing has been demonstrated to be effective in working with fungal collections (Miller et al., 2022), when the Illumina MiSeq Nano is applied to 1000 fungarium specimens, one will theoretically achieve up to 800 reads for each specimen at a sequencing cost between $1–2 per specimen (Appendix S1).

Novel HTS approaches have previously been used in the study of natural history collections (e.g., Forin et al., 2018; Folk et al., 2021). While several high‐throughput approaches have been developed specifically for specimen DNA barcoding (Shokralla et al., 2014, 2015), and even for fungal herbarium tissue in some cases (Gueidan et al., 2019; Miller et al., 2022; Runnel et al., 2022), the modified metabarcoding approach presented in this study was developed to facilitate the multiplex sequencing of numerous museum specimens and to address the trifecta of scale, effectiveness, and cost that limit the comprehensive study of fungal collections. To assess the utility of this approach, we apply these methods to a broad sampling of fungi within the subkingdom Dikarya and test its effectiveness on older specimens, along with its capacity to recognize MOTUs.

## METHODS

### Sampling

We sampled tissues from 766 dried specimens from several fungaria (Table 1, Appendix S2). Most specimens were sampled from the Sam Mitchel Herbarium of Fungi at Denver Botanic Gardens (DBG; Denver, Colorado, USA) and represent macrofungi from the Southern Rocky Mountain region. The specimens sampled represent diverse orders of Dikarya, as summarized in Table 1. A total of 384 specimens belong to the genus *Lactarius* Pers. (Russulales). This focused set was chosen for the purpose of evaluating whether the nrITS2 sequence data are capable of recognizing MOTUs at the species rank for a well‐sampled genus. Specimens were sampled using forceps cleaned with 70% ETOH and laboratory wipes. A tissue fragment slightly larger than a grain of rice was sampled from a sporocarp's hymenium and stored in a 1.5‐mL microfuge tube labeled with the specimen catalog number.

**Table 1:** Distribution of taxa represented in the 766 Dikarya specimens primarily from the Sam Mitchel Herbarium of Fungi at DBG. Taxa are shown divided by phylum, class, order, and Russulales genera *Russula* and *Lactarius*. Areas in gray highlight taxonomic subdivisions of the above taxa.

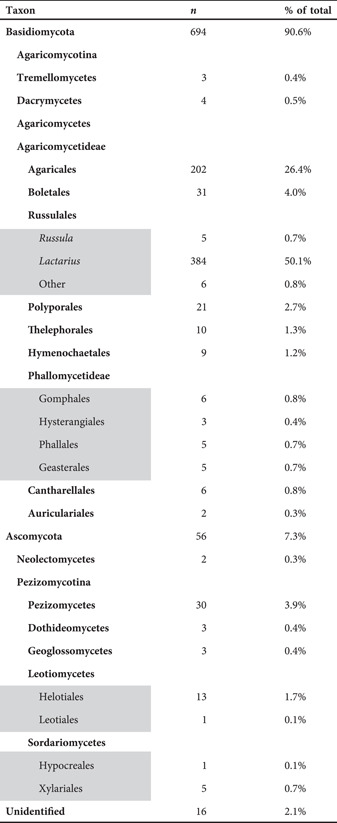

### DNA extraction

We used two methods of DNA extraction to compare a simple method designed for efficiency and cost‐effectiveness with a method designed for DNA quality. The simple method uses the alkaline extraction buffer described by Vandepol et al. (2020), with a pinch of sterile sand added to each tissue sample. The samples were left in buffer for 10 min at room temperature before being frozen to soften the tissues (temperatures <0°C will suffice for freezing). The samples were then thawed and ground until homogenized using a sterilized micropestle (catalog no. 12‐141‐368; Thermo Fisher Scientific, Waltham, Massachusetts, USA), before being incubated at 90°C for 10 min. Next, 200 µL of 3% bovine serum albumin (BSA) solution was added to each sample, which were then vortexed and centrifuged briefly (30 s) to concentrate denser material at the bottom of the tube. The samples were then considered PCR ready, with DNA templates for PCR drawn directly from the supernatant. The second DNA extraction method followed the materials and instructions provided in the E.Z.N.A. Fungal DNA Mini Kit (Omega Bio‐tek, Norcross, Georgia, USA; hereafter referred to as the mini kit extraction method).

### Sanger sequencing

We performed Sanger sequencing to compare with the HTS method. The PCR was performed in 25‐µL volumes: 1.25 µL each of the forward primer ITS1F (Gardes and Bruns, 1993) and the reverse primer ITS4 (White et al., 1990), both at a 10‐pM concentration; 12.5 µL of MyTaq Mix (Meridian Biosciences, Cincinnati, Ohio, USA); 9 µL of PCR pure water; and 1 µL of DNA template. The PCR thermal cycler steps involved an initial denaturation for 2 min at 95°C; followed by 33 cycles of denaturation at 95°C for 30 s, annealing at 55°C for 30 s, and an extension at 72°C for 2 min; followed by a final extension at 72°C for 10 min; after which the PCR ended and the products were stored at 4°C. All PCR products were inspected using electrophoresis in a 1% agarose gel. Products with no visible bands were not processed further. PCR products producing strong bands were cleaned using a simple ethanol precipitation. This entailed mixing the product with one volume of 100% ETOH, vortexing to mix, then letting the sample stand for 10 min before centrifuging at maximum speed for 2 min. The supernatant was removed without disturbing the pellet, then 200 µL of 75% ETOH was added, vortexed to mix, and centrifuged at maximum speed for 30 s. The supernatant was again removed and the tubes were placed on a 65°C heat block to evaporate the residual ETOH before 20 µL of PCR pure water was added to suspend the DNA. The cleaned PCR products were quantified using a NanoDrop (Thermo Fisher Scientific). A minimum of 25 ng of DNA was sent to ELIM Biopharmaceuticals (Hayward, California, USA) for Sanger sequencing using the ITS4 primers as these produced the clearest data for the nrITS2 region.

### High‐throughput sequencing

**Figure 1:**
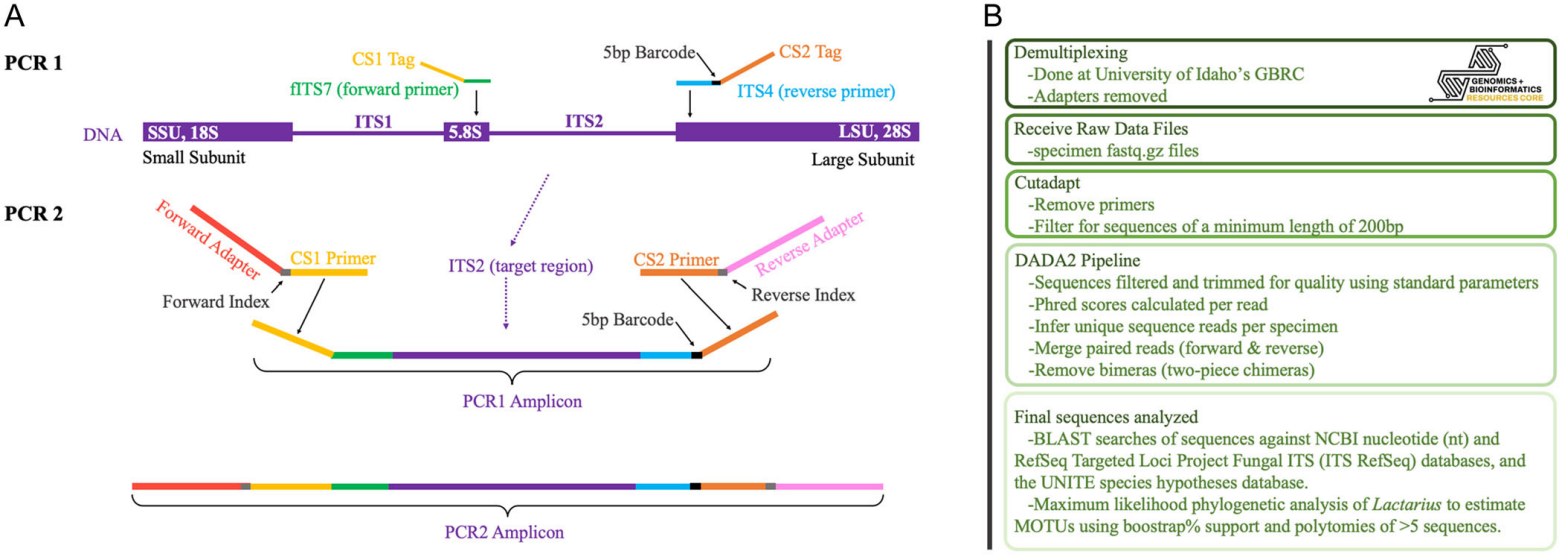
Primer map and amplicon generation for the Illumina sequencing method. (A) Two‐step PCR for Illumina sequencing. During PCR1, the forward primer fITS7 and reverse primer ITS4 target the nrITS2 region of the DNA. These primers include a 3′ CS (Fluidigm consensus sequence) tag that provides a priming site for PCR2. Between the ITS4 primer and CS tag is a 5‐bp barcode (black rectangle). During PCR2, paired‐end Illumina adapters prime to the CS tags of the PCR1 product. (B) Bioinformatic pipeline for processing the Illumina sequence reads.

We modified an Illumina MiSeq approach developed for metabarcoding of environmental fungal communities (Ihrmark et al., 2012) to sequence numerous macrofungal specimens. Our method can be described as a highly multiplexed approach, in which barcoded primers are later nested between Illumina indices in a two‐step PCR process outlined in Figure 1A. The first PCR step (PCR1) targets the ITS2 portion of the nrITS region using primers fITS7 (Ihrmark et al., 2012) and ITS4 (see above). Attached to the 5′ end of the primers is a variable spacer region followed by a 3′ CS tag (Fluidigm consensus sequence) that provides a priming site for the secondary PCR. The variable spacers consist of an additional 0–6 base pairs in the forward primer and 0–4 base pairs in the reverse primer. We modified PCR1 by inserting a 5‐bp barcode between the ITS4 region and the spacer of the reverse primer (black bar in Figure 1A). The eight unique 5‐bp barcode sequences we introduced were sourced from the work of Shokralla et al. (2014). Each DNA sample was assigned one of the eight barcoded primers. PCR1 products—each representing a sample with a unique PCR1 barcode—were pooled prior to amplification in PCR2. The PCR2 step uses primers with CS tags that anneal to the complementary sequence of the PCR1 product. Adjacent to the CS tags are unique paired‐end Illumina indices for the further multiplexing of samples, followed by Illumina adapters. We performed the PCR2 reactions on 94 pools of eight specimens each, and on two pools of seven specimens each, for a total of 766 specimens across 96 reactions. The primer sequence information for PCR1 and PCR2 is provided in Appendices S3 and S4, with the protocols for the PCRs provided on the Open Science Framework (OSF; https://osf.io/uhnxy/).

PCR1 and PCR2 use the 25‐µL reaction mixes as described above for Sanger sequencing. The thermal cycler settings for PCR1 were an initial denaturation at 95°C for 2 min; 30 cycles of denaturation at 95°C for 60 s, annealing at 54°C for 60 s, and extension at 72°C for 60 s; followed by a final extension at 72°C for 10 min. The PCR2 reaction settings were an initial denaturation at 95°C for 1 min; 8–15 cycles of denaturation at 95°C for 30 s, annealing at 60°C for 30 s, and extension at 68°C for 60 s; followed by a final extension at 68°C for 5 min. When performing PCR2, consider lowering the number of cycles to as few as eight in order to avoid over‐amplifying the product, as this is a primary cause of PCR bubbles (https://support.illumina.com/bulletins/2019/10/bubble-products-in-sequencing-libraries--causes--identification-.html).

We evaluated the PCR1 and PCR2 amplifications using electrophoresis on a 1% agarose gel. The PCR1 products were assessed into one of three categories: strong, weak, or no apparent amplification. All PCR1 products were cleaned using the same ethanol precipitation method described above, with modifications for the 0.2‐mL wells in 96‐well plate format. The barcoded PCR1 products were multiplexed into pools of eight unique barcoded samples. Each PCR1 product was sampled for pooling in 3‐µL, 5‐µL, or 10‐µL volumes based on their gel electrophoresis assessments of strong, weak, and no apparent amplification, respectively. This was done to streamline the normalization of hundreds of PCR products within a sequencing pool or library. The PCR2 products were then pooled into a single library and sent to the University of Idaho's IIDS GBRC. There, the library underwent a fragment analysis, qPCR, and bead‐based size selection and library amplification before being sequenced using an Illumina MiSeq V2 (Nano 500 cycles: 2 × 250‐bp paired‐end sequencing).

### Sanger sequencing data analysis

Sequence data received as .abi files were read and edited using default parameters for Geneious version 2020.2.2 (https://www.geneious.com). All sequences were trimmed from the ends, removing sequence data with poor electropherogram signals. Contigs were assembled if forward and reverse reads were available for a specimen, except where specimens were sequenced only in the reverse direction (ITS4) to target the ITS2 region. Simple BLAST queries through the National Center for Biotechnology Information (NCBI) GenBank nucleotide (nt) database were used to evaluate the taxonomic congruence between the specimen and the sequence, as determined using the best match.

### Illumina sequencing data analysis

Sequence data were received from the GBRC already de‐multiplexed by specimen and with the 5′ primer and spacer sequences removed (Figure 1B). The evaluation of these data begins with the removal of overhanging primers, spacers, and adapters from the 3′ ends of reads using Cutadapt (Martin, 2011), discarding reads shorter than 200 bp after trimming. The sequences were next run through the DADA2 pipeline (Callahan et al., 2016), following the software's recommended workflow as detailed below. DADA2 supports single‐nucleotide resolution analyses with detailed error and quality‐filter handling, an approach that fits the goal of precisely barcoding individual specimens while taking advantage of the sophisticated existing tools for processing community amplicon libraries.

To trim and remove low‐quality reads, the DADA2 filterAndTrim function was run with the maximum expected error per read parameter set to 3. This was followed by running the DADA2 denoising steps, first modeling a sequence error distribution then applying that model to correct likely erroneous reads in the data set. Corrected forward and reverse reads were merged where possible to produce full PCR fragment sequences. Bimeras (two‐part chimeras) were removed based on fragment distributions using the per‐sample chimera detection option. The final product of this pipeline was a set of high‐quality sequence variants detected in each sample, along with the number of reads supporting each variant.

The number of read pairs obtained per specimen was used as one metric of the success of a specimen's sequencing and the overall multiplexing approach, as well as to assess read processing at each step. We examined the number of read pairs per specimen both from the raw data files before processing and after the quality‐control processing with DADA2. This enabled us to determine whether there was a correlation between a successful or apparent PCR1 amplification and sequencing as revealed by the number of read pairs (before and after processing) and the quality scores from a specimen's read pairs.

The distribution of unique nrITS2 sequences was evaluated for each specimen. In a traditional environmental metabarcoding sequencing study, DADA2 variants represent different sequences detected in an ecological sample (or set of samples); here, different variants represent distinct unique sequences found in a single specimen's PCR fragment pool. While more than one unique sequence can be present in the processed Illumina data for most specimens, one sequence consistent with the specimen's DNA barcode is expected to be more abundant (>2× the number of reads) than other variants within the specimen's read pool. From this rationale, the sequence supported by the greatest number of reads for each specimen was selected as the representative nrITS2 DNA barcoding sequence for the downstream analysis. In rare instances of ties, the first DADA2 output sequence was used arbitrarily for this representative. The other sequence variants detected in a specimen's Illumina data pool include intragenomic variants (e.g., alleles), contaminants, and potential erroneous sequences that slipped through processing; many of these are at very low abundance in the specimen read pools.

### Sequence evaluations

Representative sequences from the Illumina and Sanger methods were evaluated for the taxonomic correlation of their specimens using BLAST searches of the NCBI nt, NCBI RefSeq Targeted Loci Project Fungal ITS (ITS RefSeq) (Schoch et al., 2014), and UNITE SH (Kõljalg et al., 2005) databases. The proportions of sequences that fell within the categories of 97.5–100%, 95–97.5%, 90–95%, 85–90%, and <85% sequence similarity with the top hit in each database were compared to assess the existence of similar sequences to the existing databases, for both the more inclusive but less reliable NCBI nt collection and the well‐curated but more sparsely populated ITS RefSeq collection. The broader taxonomic rankings across all sequences were evaluated using the UNITE species hypothesis matching analysis tool (https://github.com/TU-NHM/sh_matching_pub) as implemented using the free online PlutoF data management platform (https://plutof.ut.ee; data downloaded 10 June 2021) with the ITS2 option and otherwise default settings.

### Phylogenetic analyses

A DNA matrix of *Lactarius* sequence data was generated from the Sanger and Illumina sequencing methods. Sequence data from the top BLAST hits of the NCBI nt, ITS RefSeq, and UNITE databases were also added to the data set. Because this data set represented Rocky Mountain species of *Lactarius*, we included at least one representative ITS sequence from each species identified by Barge and Cripps (2016). The initial auto‐alignment was performed using the MUSCLE version 3.8.31 alignment tool (Edgar, 2004). Additional manual adjustments were performed using JalView (Waterhouse et al., 2009). The phylogenetic analysis was performed in RAxML (Stamatakis, 2014) with the ‐HPC2 on the XSEDE version in the CIPRES Science Gateway (Miller et al., 2010), using preset parameters. A rapid bootstrapping analysis was performed using 1000 bootstrap replicates. The trees were visualized using FigTree version 1.4.4 (Rambaut and Drummond, 2019). The phylogeny was used to recognize whether nrITS2 sequence data could reliably form MOTUs represented by multiple specimens. These MOTUs were defined as clades with more than three sequences, representing specimens with the same species identification, and with bootstrap support greater than 75%, or the highest supported polytomy with more than five sequences.

## RESULTS

All data, raw and processed, as well as detailed protocols, are available on a project page for this study through the OSF (https://osf.io/uhnxy/). Bioinformatic scripts can be accessed through the OSF or through GitHub (https://github.com/garyolds/Highthroughput-Metabarcoding-of-Macrofungi).

The DNA from 337 specimens, including 42 *Lactarius* specimens, was extracted for Sanger sequencing using the mini kit extraction method, and 274 (42 *Lactarius*) were sufficiently PCR‐amplified to be sent for sequencing. Of the completed sequences, 54 were too low quality (e.g., bad electropherogram signal) or were too short after trimming and were therefore discarded. This left 220 sequences (80%) remaining for analysis.

**Figure 2:**
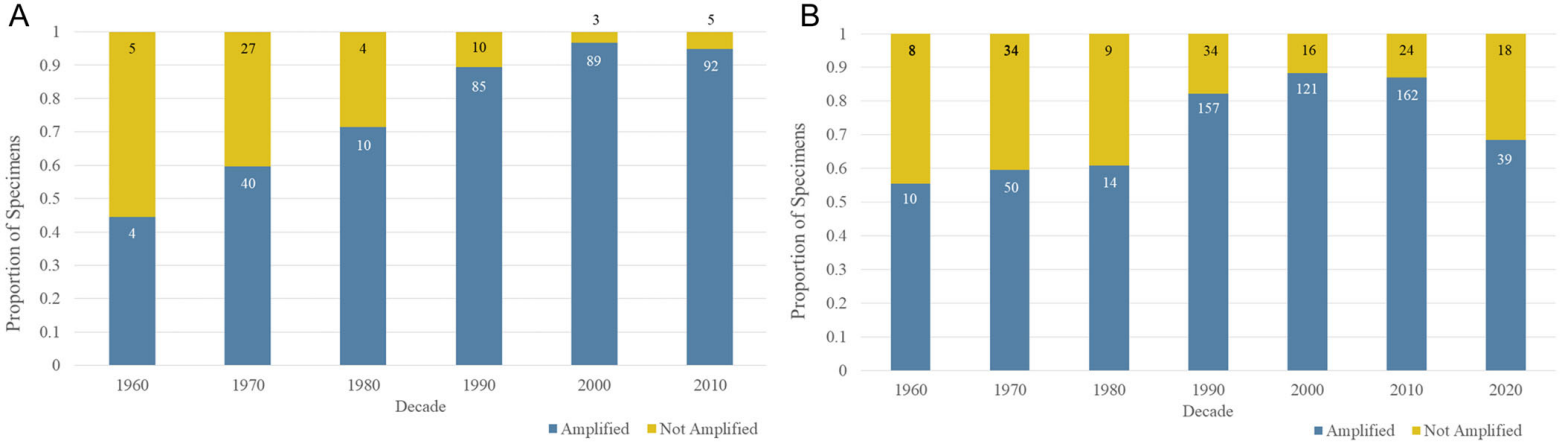
PCR1 nrITS2 amplification success rates. (A) PCR1 success rates for the 382 *Lactarius* specimens. (B) PCR1 success rates for all 766 macrofungal specimens divided by decade. Sample sizes are annotated as numbers in the amplified/non‐amplified portions of each column. Specimens lacking date information were excluded.

For the Illumina sequencing, 474 specimens were extracted using the simple extraction method and 292 specimens were extracted using the mini kit extraction. The gel electrophoresis evaluation of PCR1 revealed no discernable difference in amplification success related to the extraction method; 136 specimens did not appear to amplify, 203 were weakly amplified, and 427 were strongly amplified. As expected, the age of the specimen was the most important predictor of PCR1 success, with a greater proportion of older specimens appearing to fail PCR amplification overall (i.e., no band in the gel; Figure 2). Regardless of PCR success, all 766 specimens were processed for PCR2. All 96 pools of PCR2 reactions produced amplifications, but because nearly every reaction contained a DNA template pool of seven to eight PCR1 samples, the amplification appeared as multiple faint bands or smears on the gel.

**Figure 3:**
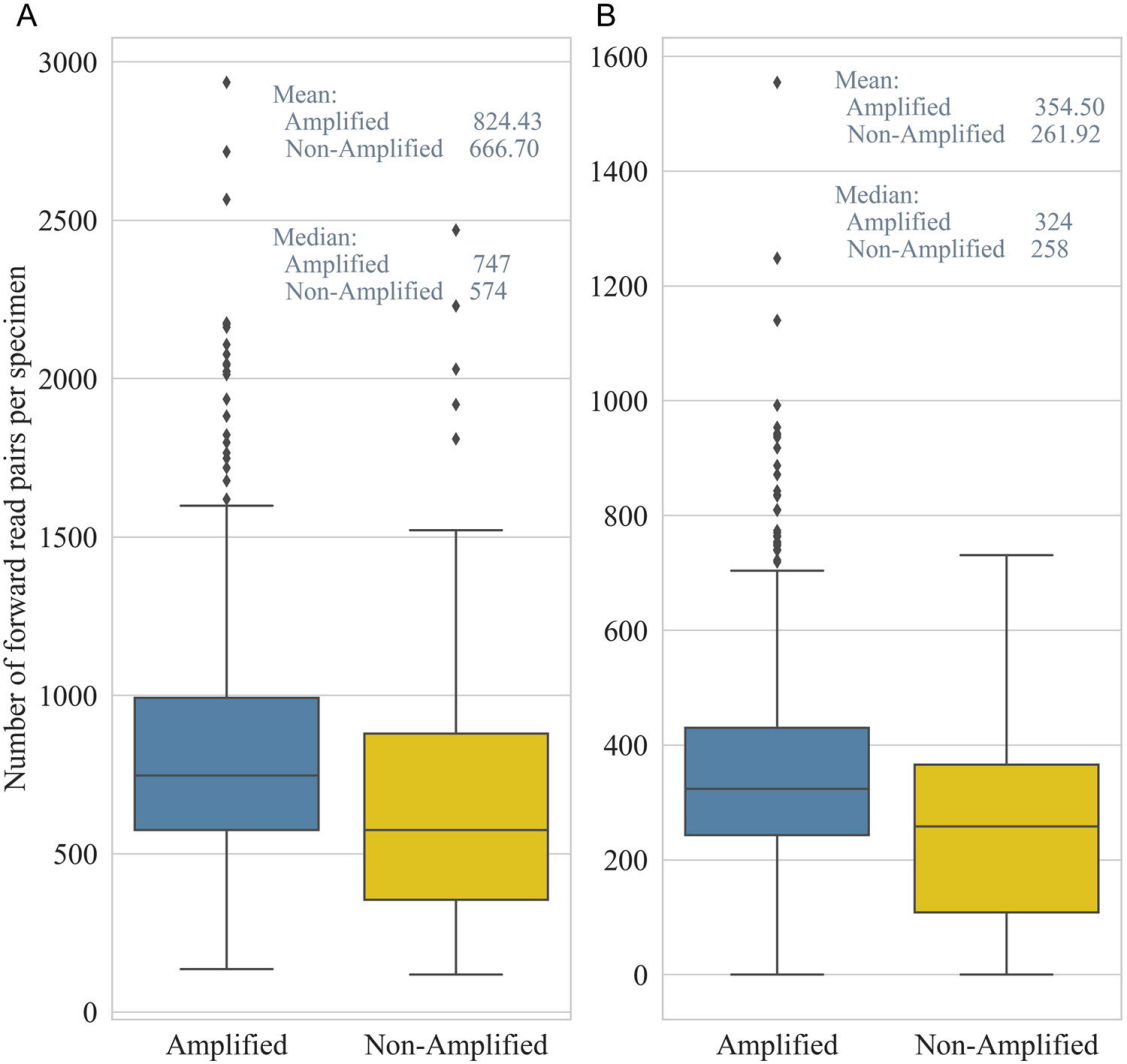
Distribution of forward reads per specimen relative to PCR amplification success. (A) Number of reads from the raw data. (B) Number of reads after DADA2 quality filtering of the data. Specimens that visibly PCR‐amplified with strong or weak bands are in blue, and those that did not appear to amplify are in yellow.

When assessing the relationship between PCR success and sequencing success from the raw data, the specimens that did not appear to amplify resulted in fewer reads than the specimens that were visibly PCR‐amplified (Figure 3A). Despite this, the specimens producing no bands from PCR1 still had an average of 666 forward reads, and there was significant overlap in whisker plots representing 95% of the distribution. Similar relative read proportions were maintained between the specimens that appeared to be PCR amplification successes or failures after the quality assessments using DADA2 filtering and the removal of short reads (Figure 3B). Even though the total number of quality reads was reduced by more than 50%, the means for specimens showing positive and negative PCR amplifications were 355 and 262 reads, respectively. Each mean falls within the interquartile range of the other category, and the whiskers for each plot overlap by nearly 100%. The Phred score mean (37.44) and median (38) at the end of quality assessment were almost identical between the specimens that were successfully PCR‐amplified and those that were not (Appendix S5).

The mean number of read pairs per specimen was 790.2 in the raw data, decreasing to 334.4 read pairs per specimen after the quality assessment was completed (Appendix S6). Before the removal of poor‐quality forward and reverse reads from the raw data files, the mean Phred score was ≤36.5 (Appendix S7). After the poor‐quality reads were removed, the mean forward and reverse read Phred scores increased slightly to 37.44 and 37.26, respectively.

**Figure 4:**
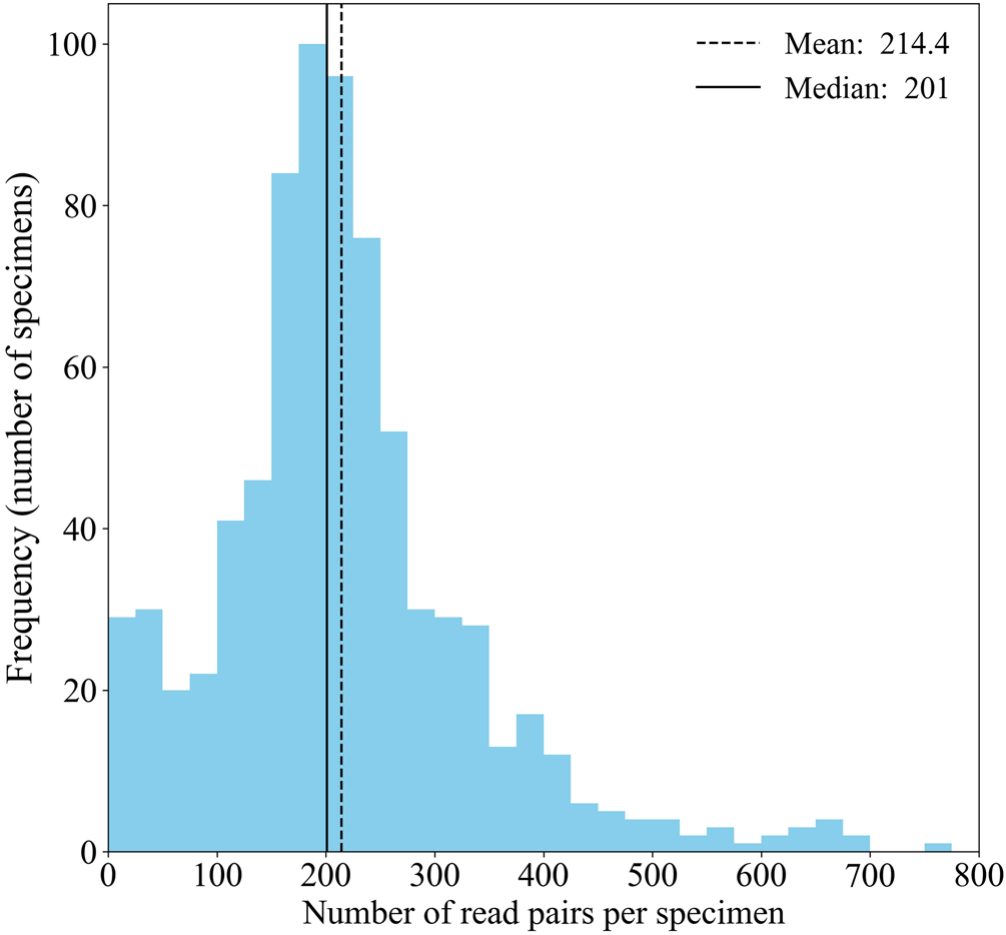
Distribution of the number of contigs (read pairs) representing the most abundant recovered from each of the 762 specimens.

Four of the 766 specimens failed to produce any sequences at the end of the DADA2 processing and quality‐filtering pipeline, representing a failure rate of 0.5%. Three of these four specimens had not appeared to amplify during PCR1. This indicates that, of the total 136 specimens that did not appear to amplify, 133 still produced usable sequence data. When data from the Sanger and Illumina methods were compared, Sanger produced 220/274 (80%) usable sequences, while the modified metabarcoding method produced 762/766 (99.5%) sequences (Appendix S8). In total, sequence data from 762 specimens had read pools that not only passed quality filtering but were also large enough to select a representative sequence after overlapping contigs of the forward and reverse reads were created. The most abundant sequence in a specimen's read pool had a mean of 214.4, with a median of 201 reads (Figure 4).

**Figure 5:**
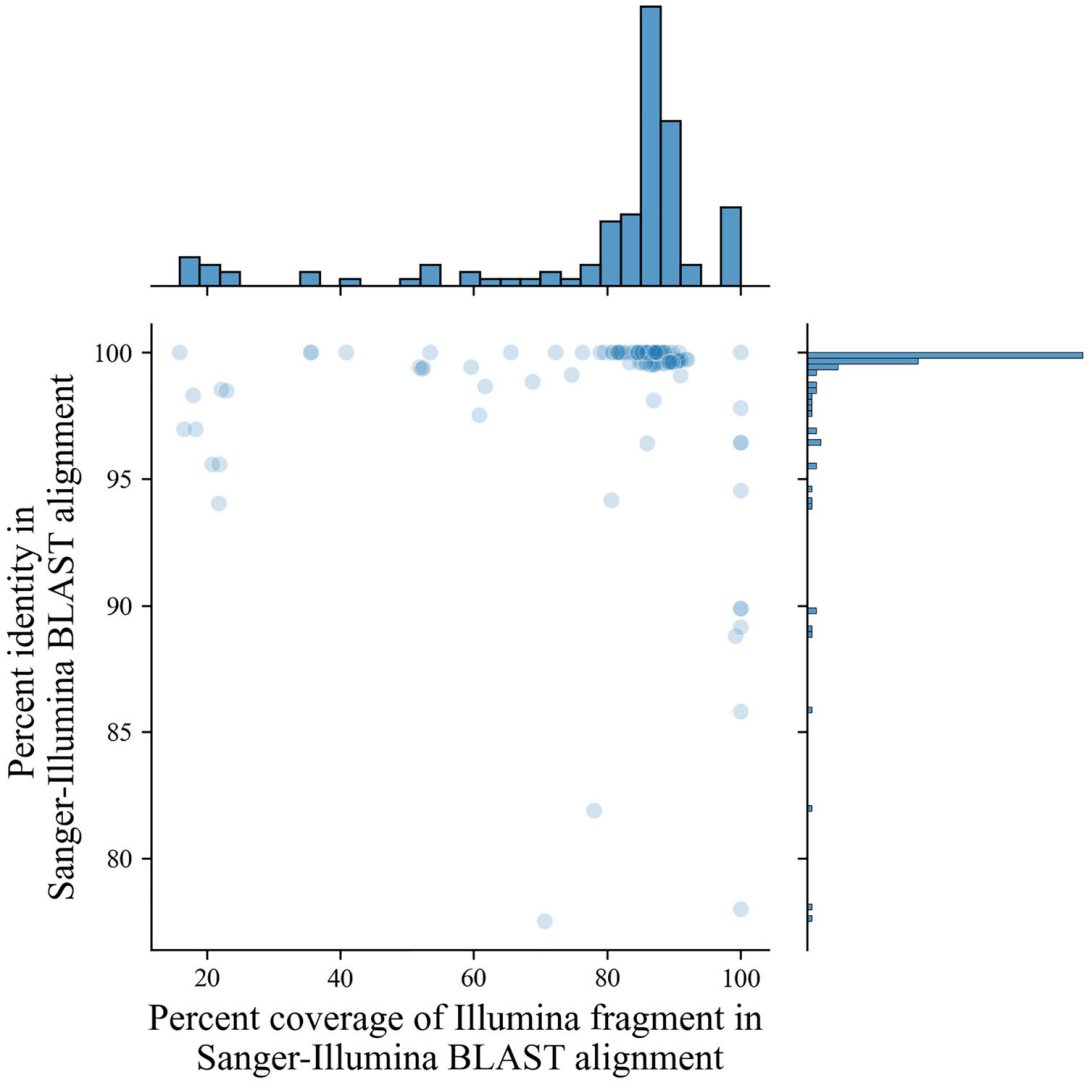
Percent identity match and overlap of the Sanger–Illumina sequence BLAST alignment. All Sanger‐sequenced specimens were aligned with their Illumina sequence using BLAST. Most specimens aligned at a near‐100% identity match, with trimmed Sanger sequences covering over 80% of the Illumina sequences when comparing from the same specimen.

To test whether the Sanger sequencing data matched the Illumina data from the same sequences, BLAST alignments were created and the percent identity (percentage of matching nucleotides in the aligned region) and percent coverage were compared between sequencing methods. The results demonstrate a high correlation of percent identity between the Sanger and Illumina sequences in the BLAST alignments (Figure 5). In addition, over 80% coverage was observed between these sequence alignments. The coverage in the Sanger–Illumina comparisons was less than 100% due to the trimming of the Sanger sequences adjacent to the ITS4 primer, resulting in shorter sequences than the Illumina sequences, which span the entire region between the primers.

**Figure 6:**
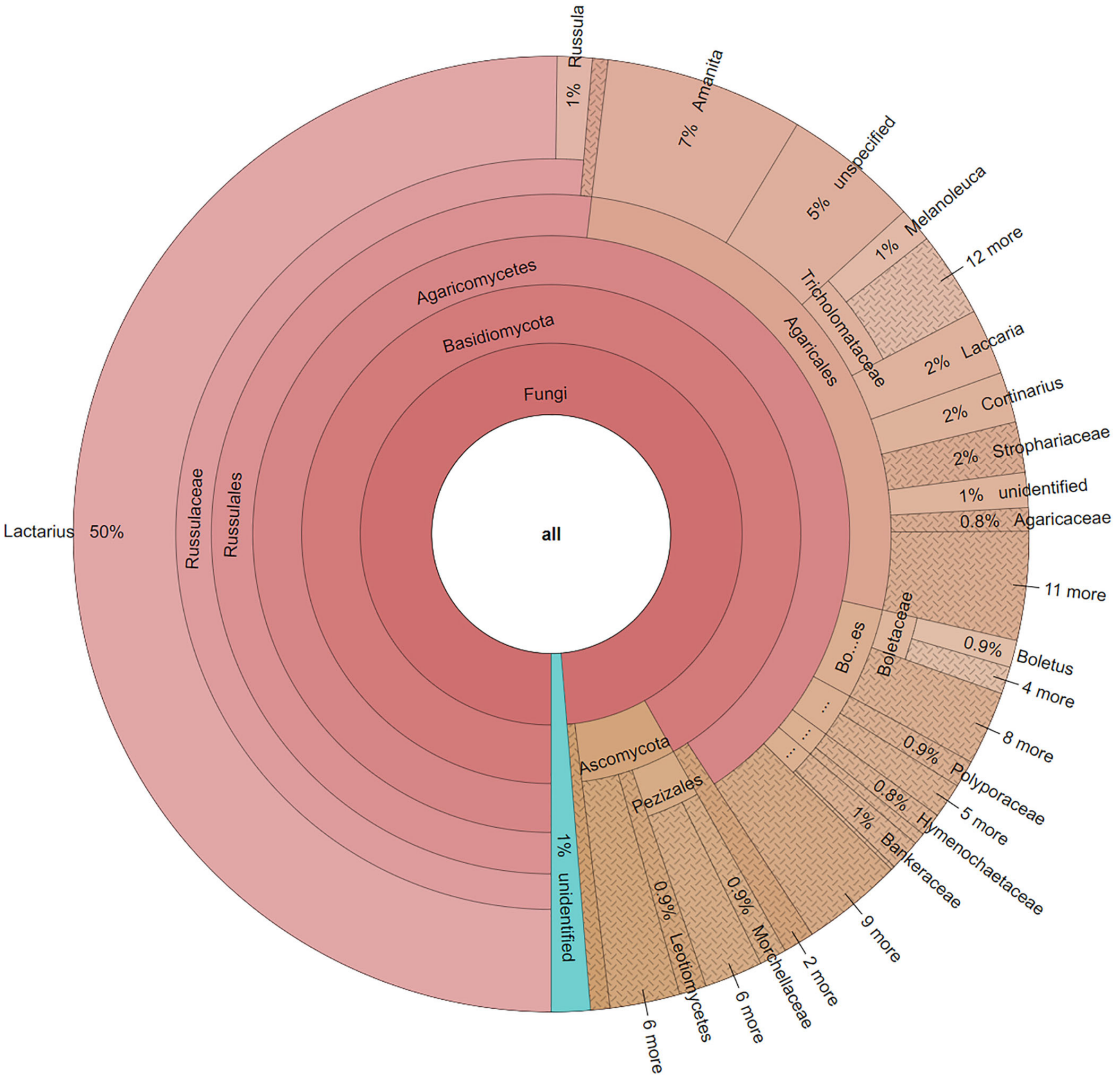
UNITE species hypotheses found in the 762 ITS2 Illumina sequences. Matches were produced using a development version analysis in the PlutoF data management platform (https://pluto.ut.ee).

In testing the applicability of these methods across taxonomic groups in the Dikarya, Figure 6 shows the distribution of data as presented in the PlutoF data management platform, which was found to broadly match the proportion of specimens sampled for this study (Table 1). To further evaluate the results of the Illumina sequence data, we used GenBank and UNITE to investigate species hypotheses and explore the taxonomic identities. The results of the percent similarity and percent overlap to the top sequence match from each of these databases are provided in Figure 5. The Illumina sequence data produced from the macrofungal specimens had varying representations across databases, with the NCBI nt database having the highest scoring hits and the ITS RefSeq database having the lowest scoring hits (Appendix S9). Upon closer evaluation of the taxonomic identities returned from the searches of the reference databases, 87 of the 766 results were not consistent with the taxonomic identification noted on the specimen. Of these, 28 could be attributed to specimen misidentification or nomenclature changes not reflected in the specimen or sequence record. The remaining 59 sequences (8% of all specimens) that did not reliably match the specimen's current identity are likely contaminants, as user error (mislabeling or switching samples) was ruled out following an investigation. Of these apparent contaminants, 48 were from material that amplified during the PCR and 39 came from specimens that had no apparent amplification. This represents 8% and 29% of these categories, respectively, indicating that relatively more contaminated sequences were derived from material with no apparent amplification. Accession numbers for all sequences uploaded to GenBank are provided in Appendix S2.

**Figure 7:**
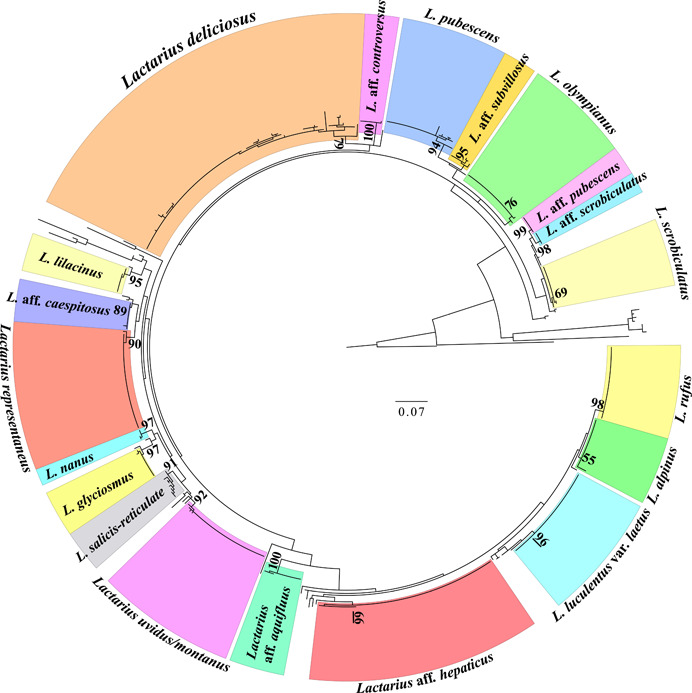
Maximum likelihood RAxML‐NG phylogeny of the 487 *Lactarius* ITS2 sequences. Newly generated sequences comprise 378 from the Illumina MiSeq Nano and 28 from the Sanger sequencing methods. The remaining 82 sequences are data from GenBank representing Rocky Mountain *Lactarius* taxa. A total of 20 clades are represented by four or more sequences and are identified as molecular operational taxonomic units (MOTUs). This is based on the resolution, bootstrap support, and the proportion sequences from named specimens within a clade. The numbers next to the branches indicate the maximum likelihood bootstrap percentages from 100 replicates.

The phylogenetic analysis of *Lactarius* ITS2 sequence data generated in this study used a data matrix of 487 sequences. In total, 377 sequences were produced using Illumina MiSeq, 28 were produced using Sanger, and 82 were sourced through GenBank (Appendix S2). The phylogeny resulted in approximately 20 *Lactarius* MOTUs from the Rocky Mountain region (Figure 7). Of these, 17 have maximum likelihood bootstrap support >75%. Two of these have 100% bootstrap support, five have 95–99%, seven were in the 85–94% range, and two had bootstrap support of between 75% and 84%. Three had bootstrap support below 75% but above 55%, which were labeled as *L*. aff. *hepaticus, L*. aff. *alpinus*, and *L. scrobiculatus* (Scop.) Fr. in the phylogeny. The failure to resolve these taxa may be due to the incomplete representation of taxa within each clade, or a lack of differentiation within the nrITS2 region in this part of the phylogeny. Further sequence data from other molecular markers will be needed to determine what limits this data set from providing better resolution for MOTUs.

## DISCUSSION

This project applies a nested barcode primer in a two‐step PCR environmental metabarcoding approach to produce a method for the mass sequencing of macrofungal specimens. This application addresses issues of scale, effectiveness, and cost that have been the primary limitations to large‐scale DNA barcoding of fungarium specimens. The ability to sequence more specimens will provide researchers with the opportunity to effectively study fungarium collections for systematic and taxonomic research.

The DNA extraction methods tested in this study did not differ in their ability to provide sequence data, indicating that the cost‐effective simple DNA extraction method is suitable for processing hundreds of specimens. Costs can be further leveraged when using the Illumina MiSeq Nano, which has a flat rate for sequencing that means the cost per nrITS2 sequence decreases as the number of specimens is scaled up (Appendix S1). The one caveat is that the MiSeq Nano kit we used is limited to 800,000 cycles, which will limit the number of specimens that can be included before the number of reads per specimen begins to affect sequence reliability. We therefore suggest limiting a MiSeq Nano sequencing run to 800–850 specimens. If sequencing more specimens (>1000) is desired, then a MiSeq V3 kit (capable of up to 10 million cycles) is recommended to ensure an appropriate sequencing depth is obtained.

The high‐throughput sequence data demonstrated several advantages over Sanger sequencing. One example was the ability to produce nrITS2 sequence data from specimens that did not appear to amplify during PCR (Figure 3). Of the specimens sent for sequencing, 99% produced nrITS2 sequence data that passed the quality‐control checks performed using the DADA2 pipeline. While 29% of these sequences turned out to be contaminants, the remaining 71% provided usable sequence data. By contrast, the specimens that did not appear to amplify after PCR were not sent out for Sanger sequencing. Of those sent for Sanger sequencing, only 80% produced usable sequence data (Appendix S8). When comparing the Illumina and Sanger sequence data, there was a near 100% match in the percent identity (Figure 5).

Addressing the 8% of sequence contamination is difficult because the source of the contamination is unclear. The potential for contamination in HTS studies is widespread (Lusk, 2014), and as a result controls are strongly encouraged for any HTS metabarcoding approach (Nguyen et al., 2015). As most of the contamination was observed in samples with no PCR amplification and as it is possible for contaminant sequences to BLAST to other macrofungi, it is possible that the contaminating sequence could have originated from amplified material from the lab. This emphasizes the importance of proper laboratory etiquette and use of controls when generating sequencing libraries in order to minimize the effects of contamination. As such, this method should be applied as a comprehensive “first pass” of large numbers of specimens to evaluate diversity in fungaria collections. To draw systematic and taxonomic conclusions, sequences should be validated through the replication and targeting of additional markers.

Our method's ability to sequence diverse lineages of Dikarya was tested in the taxonomic distribution of specimens shown in Table 1. The taxonomic diversity of sequence data represented in Figure 6 correlates to the diversity of Dikarya specimens sampled in Table 1. In evaluating the age of collections, we were able to recover sequence data from specimens collected between 1910 and 2020. The highly fragmented DNA found in older collections seems to be the Achilles’ heel for even the most advanced sequencing techniques when attempting to obtain the entire ITS region (Runnel et al., 2022). In contrast, this method, which targets a shorter section of the fungal DNA barcode, has broad application for fungaria specimens representing both taxonomic and temporal diversity.

The phylogenetic analysis of 487 *Lactarius* ITS2 sequences was considered effective in producing MOTUs. A total of 20 clades were represented by four or more sequences, 17 of which have maximum likelihood bootstrap percentages >75% (Figure 7). Of the three clades with bootstrap support <75%, only the clade labeled *L. scrobiculatus* has its identity validated with sequence data from GenBank. Clades identified as *Lactarius* aff. *alpinus* (55% support) and *L*. aff. *hepaticus* do not contain independent GenBank sequences so the MOTUs recognized by these clades are solely provided from DBG specimen identifications. Although these clades are not well supported, the genus *Lactarius* from the Southern Rocky Mountain region will be the subject of further systematic and taxonomic study using morphology and additional sequence data.

The nrITS region is considered a valuable tool in generating MOTUs in fungi when used to produce species hypotheses (Kõljalg et al., 2013; Osmundson et al., 2013). Regardless, it is important to understand the limits of the nrITS region in resolving taxonomic and systematic problems (Lindner and Banik, 2011; Hilário et al., 2021), particularly when using only a portion of the region to study diversity questions in fungi.

The flood of fungal sequence data from environmental metabarcoding studies has expanded our understanding of fungi in the environment, as well as highlighting the number of unknown fungal taxa (e.g., “dark taxa”) (Tedersoo et al., 2017; Ryberg and Nilsson, 2018). This problem is exacerbated by the fact that over 70% of fungal species do not have a representative sequence available in fungal sequence databases (Hawksworth and Lücking, 2017). Sequence data from fungarium specimens can resolve some of the issues of undescribed environmental diversity by enhancing sequence representation from vouchered specimens in sequence databases. The potential for fungarium specimens to be used to identify unknown fungi from environmental DNA samples has been demonstrated in a study of *Mortierella* Coem. (Nagy et al., 2011).

The results of this study demonstrate the potential for filling in the gaps of the fungal tree of life by DNA barcoding large numbers of fungarium specimens. The ability to sequence all the specimens of a single taxon within a collection has the benefit of resolving misidentified specimens, identifying cryptic species, and clarifying the exact nomenclature of clades by sequencing type specimens. In their study of the genus *Morchella* Dill. ex Pers., O'Donnell et al. (2011) demonstrated the significance of cryptic species in macrofungi, and similar studies have also shown cryptic diversity in North American *Cantharellus* Adans. ex Fr. (Buyck and Hofstetter, 2011; Foltz et al., 2013; Leacock et al., 2016). Access to fungarium specimens allows researchers to explore continuously evolving questions in taxonomy, and even extends the geographic coverage of taxa as it can be costly or even impossible to access historical locations to obtain fresh material. The ability to generate sequence data more broadly in fungal collections will help advance the use of fungaria in filling in the gaps in our knowledge of fungal diversity.

Lastly, this approach provides a new opportunity to address the DNA barcoding gap present in fungi. When evaluating the nrITS sequences of two species, it is necessary to produce sufficient data to understand both inter‐ and intraspecific variation. The ability to sequence multiple specimens of a taxon provides an opportunity to understand the suitability of the ITS2 region as a proxy for the fungal DNA barcode. Even though increased sampling has been demonstrated to blur the boundaries of the barcoding gap (Meyer and Paulay, 2005; Wiemers and Fiedler, 2007), the ability to sequence the DNA barcode for an entire collection helps to address the problem of undersampling the diversity among available specimens. As a result, this method improves the exploration of fungal diversity in a variety of contexts using natural history collections.

## AUTHOR CONTRIBUTIONS

C.G.O., A.W.W., and J.J.L. performed all the experiments. C.G.O. and R.B.‐T. coordinated and performed analysis on sequence data with A.W.W. performing the phylogenetic analysis. R.A.L. and A.W.W. coordinated data storage and management. A.W.W., C.G.O., and R.B.‐T. wrote the manuscript. A.W.W. conceived of the project and provided the bulk of the research support apart from that noted in the Acknowledgments. All authors approved the final version of the manuscript.

### Open Badges

This article has earned an Open Data badge for making publicly available the digitally shareable data necessary to reproduce the reported results. The data are available at https://doi.org/10.17605/OSF.IO/UHNXY.

## Supporting information


**Appendix S1**. Cost comparison between Illumina and Sanger sequencing for sequencing DNA barcodes from fungal specimens. Sanger sequenc­ing costs are based on a rate of $4.00 USD per sequence. Illumina sequencing costs are based on a baseline cost of $1500 for Illumina MiSeq Nano (500 cycles) sequencing and additional library preparation.

**Appendix S2**. Specimen list with metadata for specimens and GenBank sequence IDs.

**Appendix S3**. Primers for PCR1. Nucleotide sequence for forward ITS7f primers, spacer, CS1 adapter, and reverse ITS3 primers, Shokrala barcode, spacer, CS2 adapter.

**Appendix S4**. Primers for PCR2. Nucleotide sequence for CS1 primer, Illumina index, Illumina flow cell adapter, and CS2 primer, Illumina index, Illumina flow cell adapter.

**Appendix S5**. Average Phred scores across all specimens relative to their PCR amplification success. Speci­mens that did amplify with strong or weak bands are in blue, and those that did not appear to amplify are in yellow.

**Appendix S6**. Distribution of read pairs per specimen for each of the 766 specimens at the four stages of processing. (A) Raw sequence reads and (B–D) three benchmarks during data processing (after processing with both Cutadapt and DADA2) showing the decrease in average and median number of read pairs per specimen as the lower‐quality reads are trimmed and/or cut out of the data set.

**Appendix S7**. Distribution of the average Phred scores for the 766 specimens. The average quality scores were from the raw data files (top), after Cutadapt processing (middle), and after DADA2 quality filtering (bot­tom). Distributions of all forward reads are in yellow, and all reverse reads are in blue.

**Appendix S8**. Proportion of specimens subjected to (A) Sanger and (B) Illumina sequencing methods that were successful (green) in producing sequence data, or unsuccessful (red) in that sequence data was noisy or otherwise low quality.

**Appendix S9**. Results of sequence matches to major sequence databases.

## Data Availability

All data, raw and processed, as well as detailed protocols have been deposited and stored on a project page for this study through the Open Science Framework (OSF; https://doi.org/10.17605/OSF.IO/UHNXY, https://osf.io/uhnxy/). Bioinformatic scripts can be accessed through the OSF or through GitHub (https://github.com/garyolds/Highthroughput-Metabarcoding-of-Macrofungi).
