# Supplementary material for: Applying a modified metabarcoding approach for the sequencing of macrofungal specimens from fungarium collections
Source: Appl Plant Sci. 2023 Feb 2;11(1):e11508. doi: 10.1002/aps3.11508 (PMC9934593; doi:10.1002/aps3.11508)

DOI 10.1002/aps3.11508

APPENDIX S1. Cost comparison between Illumina and Sanger sequencing for sequencing DNA barcodes from fungal specimens. Sanger sequencing costs are based on a rate of $4.00 USD per sequence. Illumina sequencing costs are based on a baseline cost of $1500 for Illumina MiSeq Nano (500 cycles) sequencing and additional library preparation.

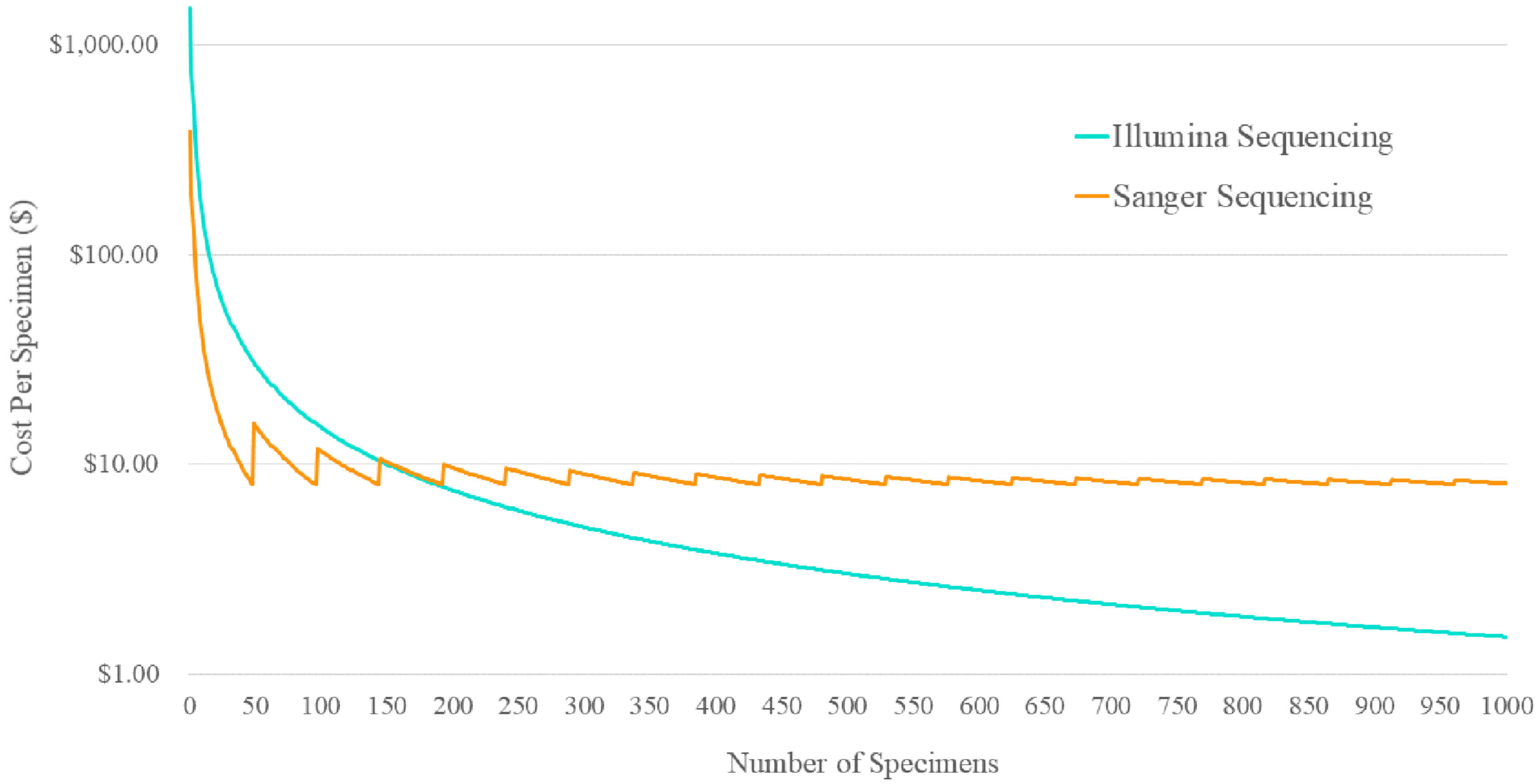

Supplement: Supplementary file 1 — Appendix S1. Cost comparison between Illumina and Sanger sequencing for sequencing DNA barcodes from fungal specimens. Sanger sequenc­ing costs are based on a rate of $4.00 USD per sequence. Illumina sequencing costs are based on a baseline cost of $1500 for Illumina MiSeq Nano (500 cycles) sequencing and additional library preparation. [file APS3-11-e11508-s001.pdf]
