# Supplementary material for: Applying a modified metabarcoding approach for the sequencing of macrofungal specimens from fungarium collections
Source: Appl Plant Sci. 2023 Feb 2;11(1):e11508. doi: 10.1002/aps3.11508 (PMC9934593; doi:10.1002/aps3.11508)

DOI 10.1002/aps3.11508

APPENDIX S5. Average Phred scores across all specimens relative to their PCR amplification success. Specimens that did amplify with strong or weak bands are in blue, and those that did not appear to amplify are in yellow.

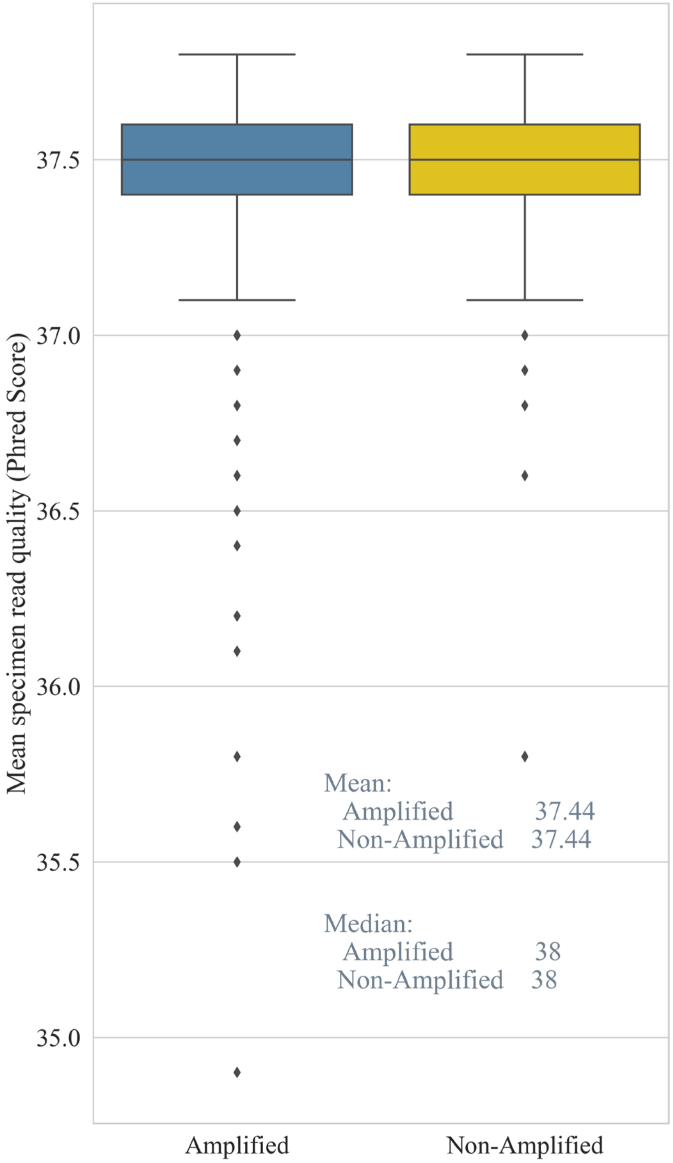

Supplement: Supplementary file 5 — Appendix S5. Average Phred scores across all specimens relative to their PCR amplification success. Speci­mens that did amplify with strong or weak bands are in blue, and those that did not appear to amplify are in yellow. [file APS3-11-e11508-s002.pdf]
