# Supplementary material for: Applying a modified metabarcoding approach for the sequencing of macrofungal specimens from fungarium collections
Source: Appl Plant Sci. 2023 Feb 2;11(1):e11508. doi: 10.1002/aps3.11508 (PMC9934593; doi:10.1002/aps3.11508)

DOI 10.1002/aps3.11508

APPENDIX S6. Distribution of read pairs per specimen for each of the 766 specimens at the four stages of processing. (A) Raw sequence reads and (B–D) three benchmarks during data processing (after processing with both Cutadapt and DADA2) showing the decrease in average and median number of read pairs per specimen as the lower-quality reads are trimmed and/or cut out of the data set.

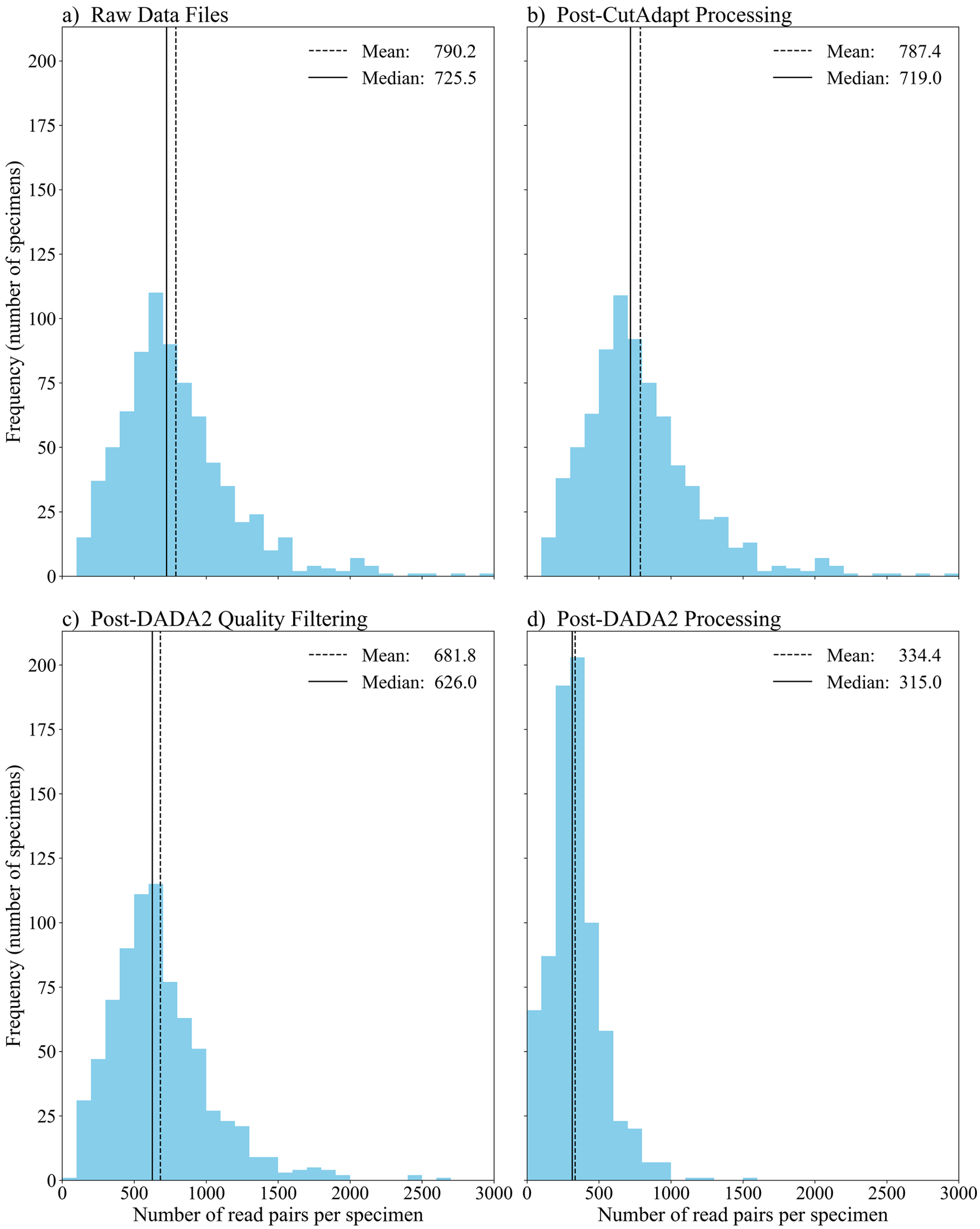

Supplement: Supplementary file 6 — Appendix S6. Distribution of read pairs per specimen for each of the 766 specimens at the four stages of processing. (A) Raw sequence reads and (B–D) three benchmarks during data processing (after processing with both Cutadapt and DADA2) showing the decrease in average and median number of read pairs per specimen as the lower‐quality reads are trimmed and/or cut out of the data set. [file APS3-11-e11508-s004.pdf]
