# Supplementary material for: Applying a modified metabarcoding approach for the sequencing of macrofungal specimens from fungarium collections
Source: Appl Plant Sci. 2023 Feb 2;11(1):e11508. doi: 10.1002/aps3.11508 (PMC9934593; doi:10.1002/aps3.11508)

DOI 10.1002/aps3.11508

**APPENDIX S7.** Distribution of the average Phred scores for the 766 specimens. The average quality scores were from the raw data files (top), after Cutadapt processing (middle), and after DADA2 quality filtering (bottom). Distributions of all forward reads are in yellow, and all reverse reads are in blue.

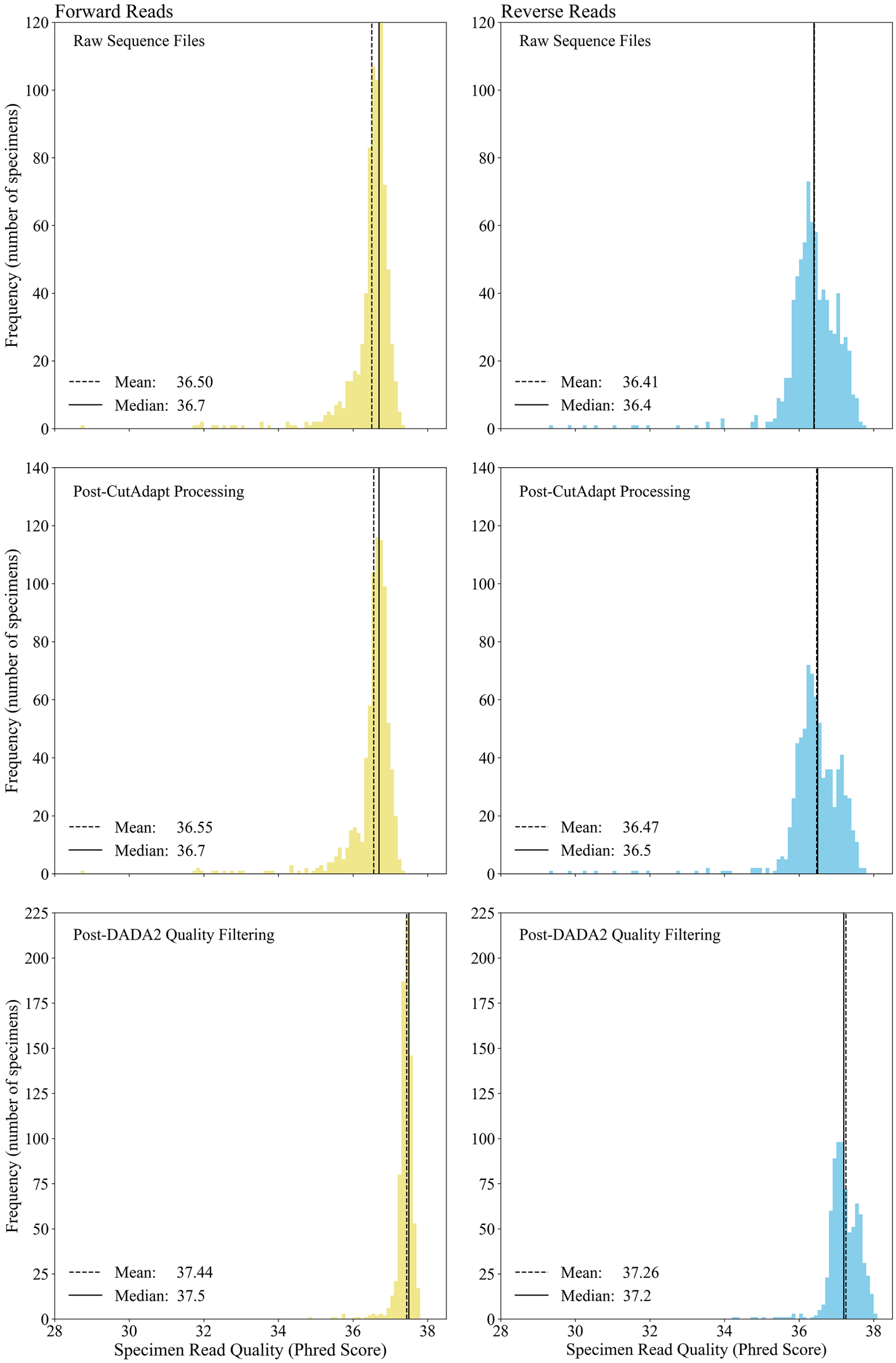

Supplement: Supplementary file 7 — Appendix S7. Distribution of the average Phred scores for the 766 specimens. The average quality scores were from the raw data files (top), after Cutadapt processing (middle), and after DADA2 quality filtering (bot­tom). Distributions of all forward reads are in yellow, and all reverse reads are in blue. [file APS3-11-e11508-s009.pdf]
