# Supplementary material for: Applying a modified metabarcoding approach for the sequencing of macrofungal specimens from fungarium collections
Source: Appl Plant Sci. 2023 Feb 2;11(1):e11508. doi: 10.1002/aps3.11508 (PMC9934593; doi:10.1002/aps3.11508)

DOI 10.1002/aps3.11508

APPENDIX S8. Proportion of specimens subjected to (A) Sanger and (B) Illumina sequencing methods that were successful (green) in producing sequence data, or unsuccessful (red) in that sequence data was noisy or otherwise low quality.

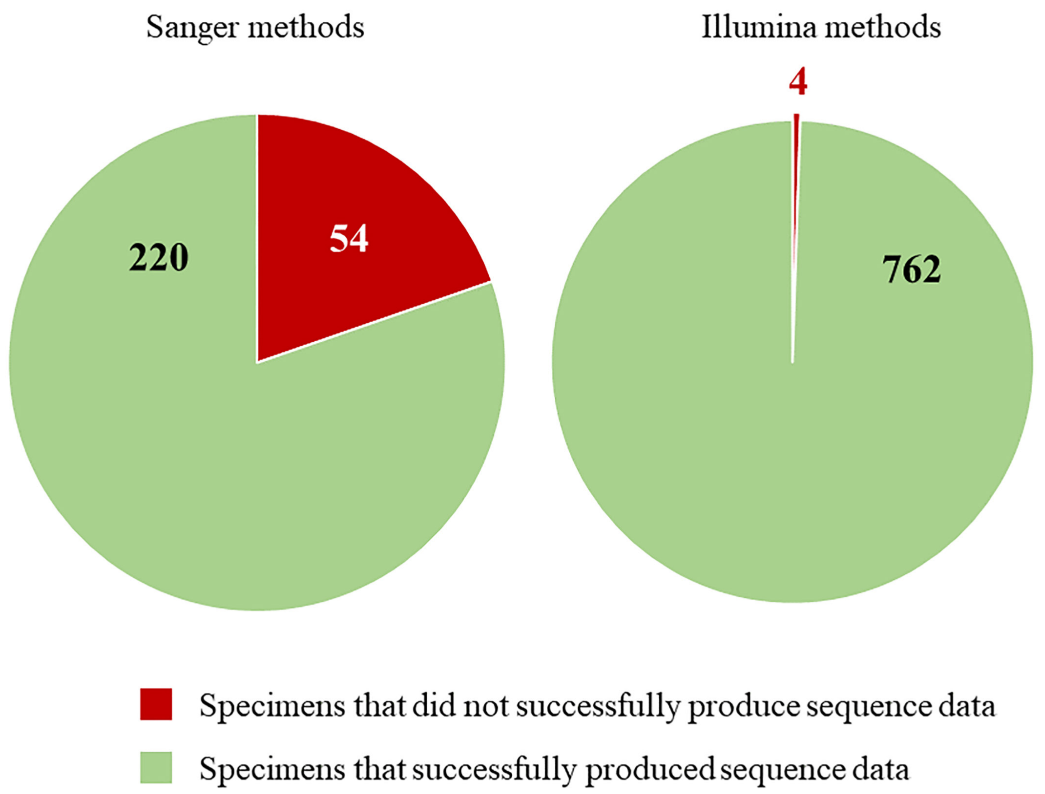

Supplement: Supplementary file 8 — Appendix S8. Proportion of specimens subjected to (A) Sanger and (B) Illumina sequencing methods that were successful (green) in producing sequence data, or unsuccessful (red) in that sequence data was noisy or otherwise low quality. [file APS3-11-e11508-s003.pdf]
