# Supplementary figures and images for: Applying a modified metabarcoding approach for the sequencing of macrofungal specimens from fungarium collections
Source: Appl Plant Sci. 2023 Feb 2;11(1):e11508. doi: 10.1002/aps3.11508 (PMC9934593; doi:10.1002/aps3.11508)

**APPENDIX S9.** Results of sequence matches to major sequence databases.

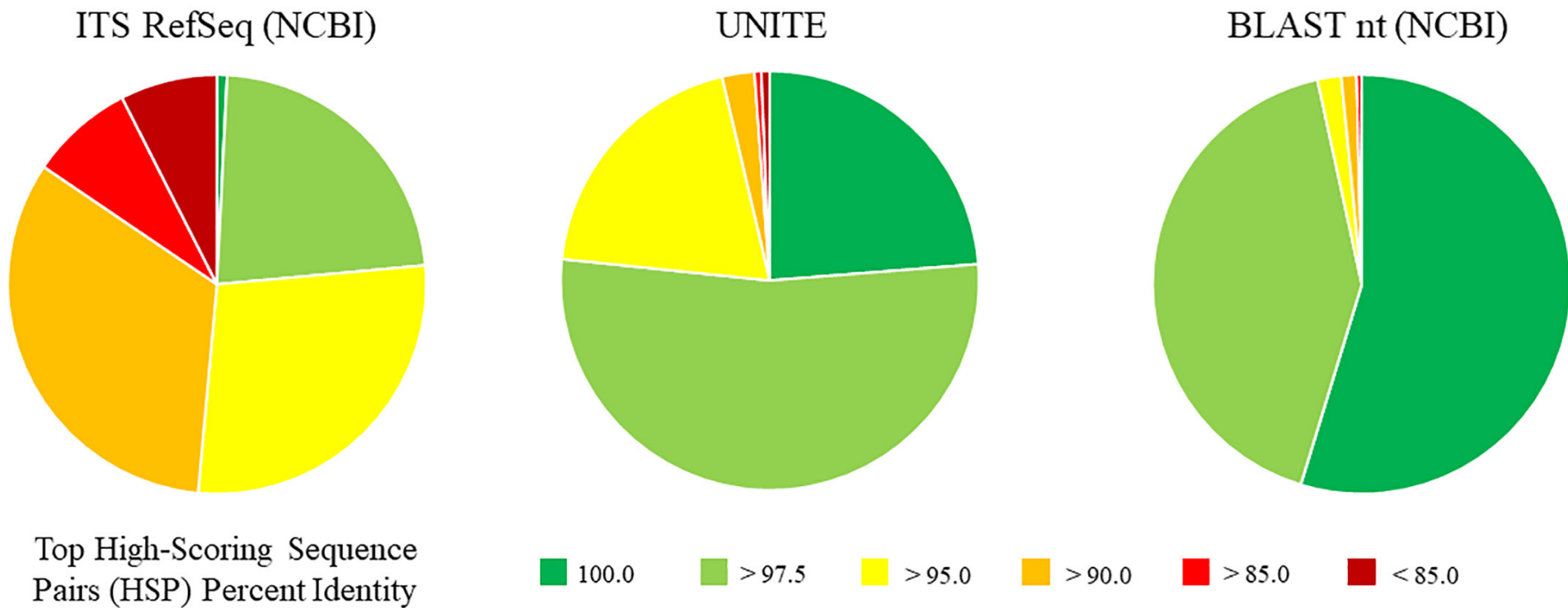

Supplement: Supplementary file 9 — Appendix S9. Results of sequence matches to major sequence databases. [file APS3-11-e11508-s008.pdf]
